# Indenopyrene and Blue-Light Co-Exposure Impairs the Tightly Controlled Activation of Xenobiotic Metabolism in Retinal Pigment Epithelial Cells: A Mechanism for Synergistic Toxicity

**DOI:** 10.3390/ijms242417385

**Published:** 2023-12-12

**Authors:** Corinne Zinflou, Patrick J. Rochette

**Affiliations:** 1 Axe Médecine Régénératrice, Centre de Recherche du CHU de Québec, Hôpital du Saint-Sacrement, Université Laval, Quebec, QC G1S 4L8, Canada; corinne.zinflou.1@ulaval.ca; 2 Centre de Recherche en Organogénèse Expérimentale de l’Université Laval/LOEX, Université Laval, Quebec, QC G1V 0A6, Canada; 3 Département d’Ophtalmologie et ORL—Chirurgie Cervico-Faciale, Université Laval, Quebec, QC G1V 0A6, Canada

**Keywords:** high-energy visible light (HEV; blue light), polycyclic aromatic hydrocarbons metabolism, indenopyrene (IcdP) phototoxicity, oxidative stress, retinal pigment epithelial (RPE) cells, aryl hydrocarbon receptor (AhR), nuclear factor erythroid-2 related factor-2 (Nrf2)

## Abstract

High energy visible (HEV) blue light is an increasing source of concern for visual health. Polycyclic aromatic hydrocarbons (PAH), a group of compounds found in high concentrations in smokers and polluted environments, accumulate in the retinal pigment epithelium (RPE). HEV absorption by indeno [1,2,3-cd]pyrene (IcdP), a common PAH, synergizes their toxicities and promotes degenerative changes in RPE cells comparable to the ones observed in age-related macular degeneration. In this study, we decipher the processes underlying IcdP and HEV synergic toxicity in human RPE cells. We found that IcdP-HEV toxicity is caused by the loss of the tight coupling between the two metabolic phases ensuring IcdP efficient detoxification. Indeed, IcdP/HEV co-exposure induces an overactivation of key actors in phase I metabolism. IcdP/HEV interaction is also associated with a downregulation of proteins involved in phase II. Our data thus indicate that phase II is hindered in response to co-exposure and that it is insufficient to sustain the enhanced phase I induction. This is reflected by an accelerated production of endogenous reactive oxygen species (ROS) and an increased accumulation of IcdP-related bulky DNA damage. Our work raises the prospect that lifestyle and environmental pollution may be significant modulators of HEV toxicity in the retina.

## 1. Introduction

Exposure to high energy visible blue light (HEV; 400–500 nm), the most energetic wavelengths normally reaching the human adult retina [1], can induce retinal injury over time through photo-oxidatively generated damage to retinal pigment epithelium (RPE) cells. It is suspected to be involved in age-related macular degeneration (AMD) [2,3,4], the leading cause of irreversible vision loss among adults over age 55 [5,6]. Dry AMD, the most common form (80–90%) of AMD, is characterized by a premature deterioration of the RPE [7,8], a multifunctional mono-layered epithelium forming the interface between the neurosensory retina and the vascularized choroid [9,10,11]. Since RPE is essential for photoreceptors’ health, its deterioration leads to progressive photoreceptor degeneration, and ultimately to vision loss [7].

The molecular basis of HEV light involvement in RPE cells deterioration is still poorly understood. Oxidative stress is suggested as a primary mechanism of HEV light toxicity for RPE cells [12,13,14,15]. Experimental evidence indicates that oxidative stress elicited by HEV light mainly relies on age-related accumulation of lipofuscin—a mixture of complex photoreactive chromophores—within RPE cells. Through photosensitized oxidation reactions mediated by lipofuscin components, HEV light promotes the formation of reactive oxygen species (ROS), eventually leading to oxidative stress and RPE cell death [12,15,16,17,18,19,20]. Cytochromes in the mitochondria respiratory chain are other proposed mediators of HEV-light-induced oxidative stress [14]. Nonetheless, we have highlighted the phototoxicity of an exogenous HEV-absorbing chromophore, indeno[1,2,3-cd]pyrene (IcdP), a lipophilic high-molecular-weight polycyclic aromatic hydrocarbon (PAH). We have shown that IcdP synergistically reacts with HEV light to induce oxidative stress, mitochondrial network collapse, and cell death in human RPE cells [21].

PAH represent a class of widespread environmental pollutants generated by the incomplete combustion of organic matter. After entering the bloodstream, they concentrate in melanin-rich tissues, such as the RPE [9,22]. RPE cells play an important role in metabolizing such xenobiotics [9,10]. Phases I (modification) and II (conjugation) metabolism are required for their elimination. With concomitant production of ROS [23,24], cytochrome P450 (CYP) monooxygenases of phase I catalyze the initial oxygenation required for further biotransformation of lipophilic PAH. Most active isoforms include PAH-inducible CYP1 members: CYP1A1, CYP1A2, and CYP1B1 [24,25,26]. PAH processing through phase II then commonly involves the conjugation activities of glutathione-*S*-transferase (GST), which produces inert excretable metabolites.

PAH modification by CYP monooxygenases results in the formation of reactive intermediates, including diol-epoxides, radical cations, or *o*-quinones, which may bind covalently to nucleic acids and proteins to form adducts [27,28,29,30]. A tight coupling between the two phases is thus crucial for limiting the detrimental effects of exposure to toxic PAH [31,32]. Coupled induction is mainly achieved through the coordinated activation of two key transcription factors: the aryl hydrocarbon receptor (AhR), a cytosolic ligand-activated transcription factor, and the nuclear factor erythroid-2 related factor-2 (Nrf2), a factor involved as a critical regulator of adaptive cell defenses against oxidative and xenobiotic stresses [32,33]. PAH binding to AhR results in AhR nuclear translocation; interaction with its dimerization partner, the aryl hydrocarbon nuclear translocator (ARNT); and formation of an active transcription factor complex [34]. The activated complex binds specific sequences, termed xenobiotic responsive element (XRE)—present in the promoter of phase I target genes—to induce their transcription. ROS and metabolites generated during phase I promote Nrf2 activation and nuclear translocation [33]. This results in its binding to antioxidant response element (ARE) enhancer sequences that mediate the transcriptional activation of phase II target genes.

Present in high levels in cigarette smoke (the most important environmental risk factor for AMD [35,36], some PAH are suspected to play a substantial part in the pathophysiological processes underlying AMD-related RPE cell defects in smokers [37,38,39,40,41]. They exhibit cytotoxic effects for RPE cells treated with PAH concentrations ranging from 50 µM to 1000 µM. In sub-lethal concentrations (10 to 200 µM), they can be genotoxic, induce alteration of RPE cells’ lysosomal and exocytotic activities, or promote a pro-inflammatory response. However, it should be noted that PAH levels accumulating in smokers’ RPE have never been investigated. But given that the estimated levels are below 200 ng/cigarette for most high-molecular-weight PAH in cigarette smoke [42,43,44,45] and that average blood concentrations for such PAH are reported between 1 ng/mL and 4 µg/mL in both smokers and people living in heavily polluted areas [45,46,47,48], we may assume that average levels of most high-molecular-weight PAH carried in the blood to the RPE are in the nanomolar range. Using some key features of AMD as an indicator of toxicity, we showed that in this range, IcdP concentrations lower than 500 nM are sufficient for triggering phototoxicity in RPE cells when it absorbs sublethal doses of HEV light [21].

In the present study, to better understand the basis of IcdP/HEV light toxic interaction, we first examined whether their synergy is a direct consequence of photosensitization reactions, mediated by IcdP acting as a photosensitizer in human RPE cells. Next, we studied the induction of the PAH metabolism in response to IcdP and HEV light co-exposure. Along with ROS, the phase I metabolism of IcdP generates some highly reactive DNA-binding species [28,29], which yield stable bulky DNA adducts. Thus, using kinetics ROS accumulation and formation of stable bulky DNA adducts as indicators of phase I activation, we assessed the impact of HEV exposure on the IcdP-triggered metabolism. We found that transcriptional modulation of the IcdP metabolism is strongly impaired by HEV light exposure. Moreover, a subcellular level analysis of AhR and Nrf2 reveals a strong disruption of the tight coupling between the phase I and phase II metabolism. Our study emphasizes a dysregulation of the IcdP-induced transcriptional response of RPE cells in the presence of HEV light, which may drive toxic synergy between both environmental agents. Metabolic impairment could thus be a mechanism of HEV light toxicity for RPE cells in the presence of pollution-derived xenobiotics.

## 2. Results

### 2.1. IcdP/HEV-Induced Oxidative Stress Is Not a Direct Consequence of Photosensitized Oxidation Reactions

**Figure 1:**
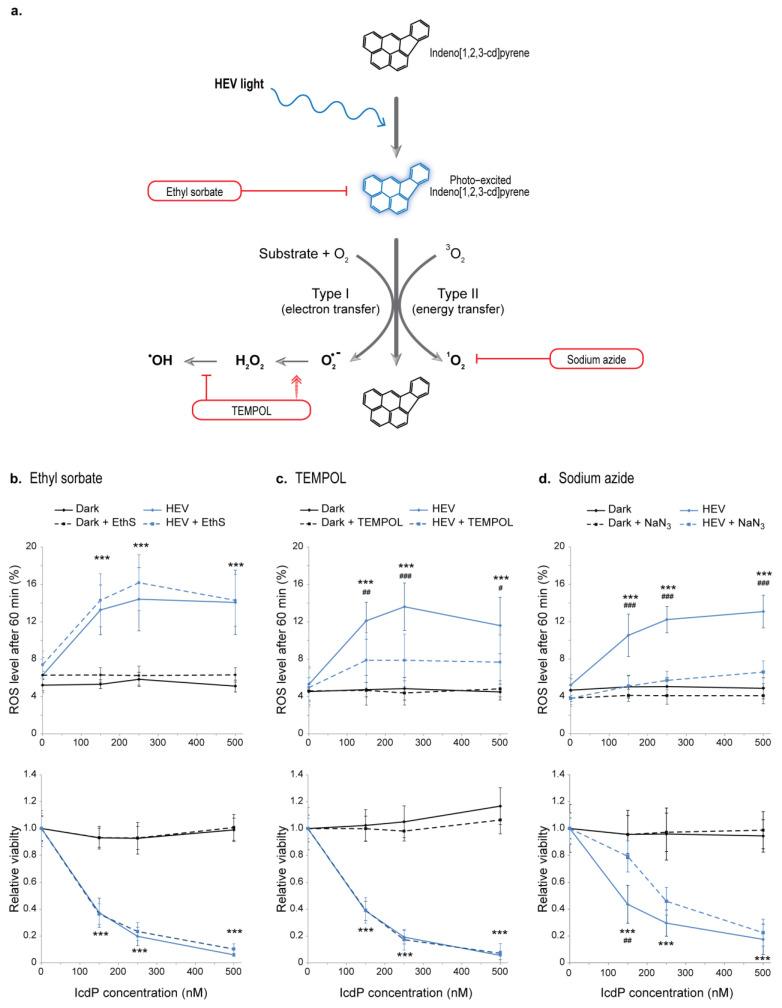
Implication of photosensitized oxidation reactions in indeno[1,2,3-cd]pyrene (IcdP) and high energy visible (HEV) light synergic cytotoxicity. (**a**) Scheme depicting IcdP-mediated photosensitized production of reactive oxygen species (ROS) under HEV light and the action of quenchers. When IcdP absorbs HEV light, it can induce ROS release via either type I or type II mechanisms. Ethyl sorbate (EthS), a quencher deactivating molecules in a triplet excited state, prevents the formation of ROS. 4-hydroxy-1-oxyl-2,2,6,6-tetramethylpiperidine (TEMPOL), a water-soluble superoxide dismutase mimetic agent, increases O_2_^•−^ conversion rate in H_2_O_2_ and prevents ^•^OH formation by inhibiting the Fenton reaction. Sodium azide (NaN_3_) specifically quenches ^1^O_2_. (**b**–**d**) Quencher or antioxidant supplementation effects on ROS accumulation (top panels) and cell viability (bottom panels) after co-exposure to IcdP and HEV light. Prior to exposure to IcdP (0–500 nM) and/or HEV light (160 J/cm^2^) (see “Materials and Methods” for details), ARPE19 cells were treated for 18–24 h with (**b**) 1 µg/mL of ethyl sorbate (EthS), (**c**) 1.5 mM of TEMPOL, or (**d**) 10 mM of sodium azide (NaN_3_). ROS accumulation was monitored for 60 min after exposure using the CM-H2DCFDA fluorescent probe (top), and cell viability was measured 24 h post-exposure using the MTS assay (bottom). Error bars are SD from at least three independent experiments (N = 3). (*) are for differences between HEV-exposed samples versus unirradiated controls and (#) are for differences between HEV-exposed samples versus HEV (+antioxidant)-exposed samples; # *p* < 0.05, ## *p* < 0.01 and ***; ### *p* < 0.001 [two-way analysis of variance (ANOVA) with Tukey’s HSD procedure as post hoc test].

ROS may arise from type I or type II photosensitized oxidation reactions mediated by IcdP under HEV light (Figure 1a). The superoxide anion (O_2_^•−^) and hydroxyl radical (^•^OH) are reactive species derived from type I reactions in biological environments, while singlet oxygen (^1^O_2_) is produced by a type II mechanism [49,50]. To investigate the implication of the two photosensitization mechanisms in IcdP/HEV- induced toxicity in RPE cells, we assessed the protective effects of ethyl sorbate (EthS, a quencher of molecules in triplet excited state), 4-hydroxy-1-oxyl-2,2,6,6-tetramethylpiperidine (TEMPOL, a superoxide dismutase mimetic agent that increases O_2_^•−^ conversion rate in low reactive H_2_O_2_ and prevents ^•^OH formation), and sodium azide (NaN_3_, a specific quencher of ^1^O_2_). ARPE19 cells, pre-treated with 1 µg/mL of EthS (Figure 1b), 1.5 mM of TEMPOL (Figure 1c), or 10 mM of NaN_3_ (Figure 1d) were exposed to IcdP (150–500 nM) and/or HEV light (160 J/cm^2^). ROS levels were then measured 60 min after exposure (top panels), and cell viability was assessed 24 h later (bottom panels).

As previously demonstrated [21], IcdP/HEV light co-exposure led to an increased ROS content in RPE cells and promotes cell death (Figure 1b–d, solid lines). We observed no protective effect from EthS pretreatment. It induces no significant changes in the amounts of ROS detected after IcdP/HEV exposure (Figure 1b, top) or in IcdP/HEV-induced loss of cell viability (Figure 1b, bottom). TEMPOL pretreatment reduces IcdP/HEV-associated intracellular accumulation of ROS (Figure 1c, top). Indeed, following IcdP/HEV co-exposure, ROS levels are 2.7, 3.0, and 2.6 times the level detected in control cells and cells treated with 150, 250, and 500 nM of IcdP, respectively. While in presence of TEMPOL, they are only 1.7 times higher than the ROS level in unirradiated TEMPOL-pretreated controls for all tested concentrations of IcdP. Nonetheless, we found no improvement of viability following IcdP/HEV co-exposure associated with TEMPOL pretreatment (Figure 1c, bottom). NaN_3_ also prevents part of IcdP/HEV-induced ROS. Without pretreatment and following co-exposure to HEV light with 150, 250, and 500 nM of IcdP, ROS are 2.3, 2.6, and 2.8 times more abundant, respectively, in co-exposed cells than in unirradiated cells. With NaN_3_ pretreatment, they are only 1.3, 1.5, and 1.7 times more abundant after co-exposure than in NaN_3_-pretreated controls (Figure 1c, top). However, 24 h after co-exposure, NaN_3_ pretreatment is significantly associated with improved viability of IcdP/HEV-exposed cells only at 150 nM of IcdP (Figure 1b, bottom). It is noteworthy that pre-treating cells with higher concentrations of EthS (up to 25 µg/mL), TEMPOL (up to 5 mM), or NaN_3_ (up to 50 mM) offers no additional protection against IcdP/HEV-induced loss of viability (Figure S1). Taken together, the data indicate that HEV-photosensitized production of ROS mediated by IcdP contributes only marginally to IcdP/HEV synergistic toxicity, and is most likely not the principal source of IcdP/HEV-induced oxidative stress.

### 2.2. ROS Generation Is Stimulated by IcdP/HEV for at Least 6 h after Co-Exposure

**Figure 2:**
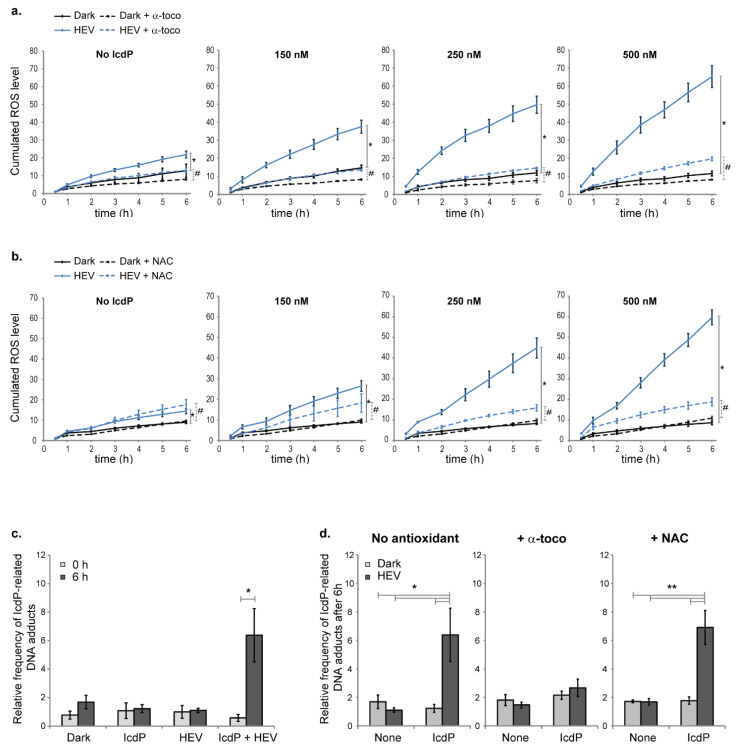
ROS and bulky DNA lesions accumulation in RPE cells following exposure to IcdP and/or HEV light. ARPE19 cells were exposed to IcdP (0–500 nM) and/or HEV light (160 J/cm^2^). (**a**,**b**) Following exposure, cumulative ROS generation patterns were assessed by monitoring intracellular ROS levels for 6 h post-exposure using the CM-H2DCFDA probe in cells treated or not treated with (**a**) 10 µM of α-tocopherol or (**b**) 5 mM N-acetylcysteine (NAC) for 16–24 h prior to IcdP and/or HEV light exposure. For cells subjected or not subjected to antioxidant pretreatment, ROS average level at 30 min in their respective unirradiated DMSO-treated controls was set as a baseline (value 1). Error bars are SD from four independent assays (N = 4); *; # *p* < 0.001 [Comparison of nonlinear regression models using F-test]. (**c**) The relative frequency of DNA polymerase-blocking bulky adducts in DNA was assessed using a modified LA-QPCR assay, immediately (0 h) or 6 h following exposure to 500 nM of IcdP and/or 160 J/cm^2^ of HEV light. (**d**) ARPE19 cells were treated with 10 µM of α-tocopherol or 5 mM NAC for 18–24 h prior to exposure to IcdP (500 nM) and/or HEV light. The relative frequency of bulky adducts in DNA was estimated 6 h following exposure. For each condition (**c**,**d**), at least three independent samples were analyzed and each sample processed in quadruplicate (n = 4, N = 3). Error bars are SEM; * *p* < 0.05, ** *p* < 0.01 [One-way analysis of variance (ANOVA) with Tukey’s HSD procedure as post hoc test].

To gain further insight on IcdP/HEV-induced oxidative stress, cumulative ROS levels were successively estimated up to 6 h post-exposure to IcdP (150–500 nM) and/or HEV light (160 J/cm^2^) (Figure 2a,b). ROS accumulate slowly over time in unirradiated control cells (Figure 2a,b, “No IcdP” panels). Their production is stimulated following HEV light exposure, with cumulated ROS produced over 6 h being 1.7 times the level cumulated in unirradiated cells. IcdP exposure alone (up to 500 nM) leads to no significant change in the dynamic of ROS buildup in unirradiated cells (Figure 2a,b, solid black lines). However, HEV light exposure of IcdP-treated cells induces a significant IcdP dose-dependent acceleration of ROS accumulation after co-exposure (Figure 2a,b, solid blue lines). The time required to accumulate an amount of ROS comparable to the 6 h ROS production in the dark is 2 h for HEV-exposed cells treated with 150 nM IcdP, and 1 h or less for those treated with 250 or 500 nM IcdP. Moreover, cumulated ROS produced after 6 h by HEV-exposed cells are 2.6, 4.2, and 5.6 times the level produced after 6 h in unirradiated cells, when cells were treated with 150, 250, and 500 nM IcdP, respectively. IcdP/HEV-induced oxidative stress is thus primarily generated after the end of exposure.

When cells are incubated with 10 μM α-tocopherol (a lipophilic broad-spectrum antioxidant) prior to the co-exposure, HEV light-related stimulation of ROS production remains unaffected, but IcdP/HEV-induced acceleration of ROS accumulation is prevented (Figure 2a, dashed lines). Indeed, in the presence of α-tocopherol, IcdP/HEV co-treated cells (up to 250 nM IcdP) do not accumulate ROS more rapidly than cells exposed to HEV light alone. Cumulated ROS levels after 6 h in HEV-exposed cells are 1.6, 1.7, 1.9, and 2.4 times the levels measured in cells kept in the dark, for cells treated with 0, 150, 250, and 500 nM of IcdP, respectively. We also tested the effect of a 5 mM *N*-acetylcysteine (NAC, a hydrophilic broad-spectrum antioxidant) pretreatment on ROS accumulation over 6 h in RPE cells following IcdP and/or HEV light exposure (Figure 2b). In the presence of NAC, final cumulated ROS levels detected in HEV-exposed cells are 1.8, 1.8, 1.6, and 1.7 times the levels measured in cells kept in the dark, when cells were treated with 0, 150, 250, and 500 nM of IcdP, respectively (Figure 2b). NAC thus appears as effective as α-tocopherol in specifically preventing IcdP/HEV-induced acceleration of ROS accumulation over 6 h post-exposure at any tested concentration of IcdP.

### 2.3. IcdP/HEV Co-Exposure Leads to the Accumulation of Bulky DNA Adducts

The formation of reactive DNA-binding species, which are likely to cause bulky DNA adducts, is an immediate consequence of phase I of PAH metabolism [27,30]. Using a modified LA-QPCR assay, we thus assessed the relative frequency of polymerase-blocking DNA lesions as an indicator of bulky DNA adducts 0 and 6 h following exposure of cells to 500 nM of IcdP and/or 160 J/cm^2^ of HEV light (Figure 2c). No blocking damage was found immediately following exposure (0 h) in IcdP-exposed cells with or without HEV light. We could not detect such damage 6 h following either IcdP or HEV light treatment alone, but 6 h post-co-exposure to IcdP and HEV, we measured blocking damage at a level almost four times above controls, indicating the presence of bulky adducts in the DNA of RPE cells (Figure 2c).

We then analyzed the effect of 10 µM α-tocopherol or 5 mM NAC pretreatment on IcdP/HEV-induced formation of bulky DNA adducts (Figure 2d). We found that pre-treating cells with α-tocopherol suppresses IcdP/HEV-induced accumulation of bulky adducts in DNA. However, no significant changes in the frequency of bulky DNA adducts is associated with NAC pre-treatment (Figure 2d).

### 2.4. Increased AhR Activation and Decreased Nrf2 Levels after IcdP/HEV Co-Exposure

**Figure 3:**
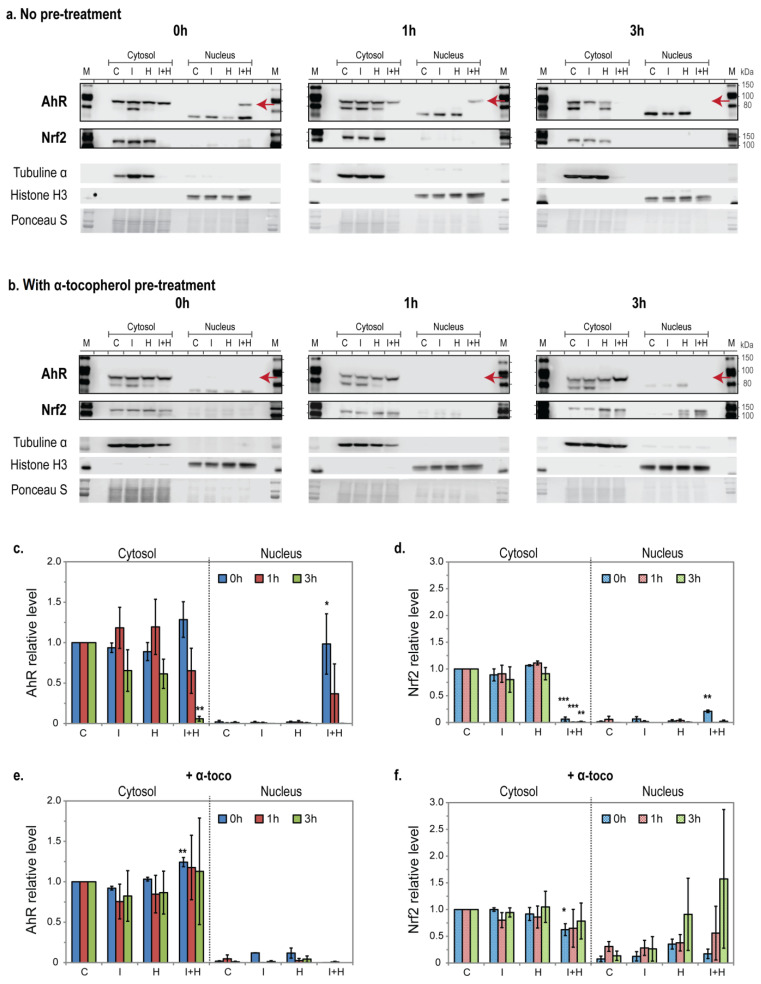
Level and subcellular localization of AhR and Nrf2 in RPE cells exposed to IcdP and/or HEV light. ARPE19 cells were exposed to 500 nM of IcdP and/or 160 J/cm^2^ of HEV light. Cytosolic and nuclear fractions were isolated immediately (0 h), 1 h, or 3 h following exposure. (**a**,**b**) Representative blots at 0, 1, and 3 h post-exposure for AhR functional form (red arrows) and Nrf2 in cytosolic and nuclear extracts from cells exposed to IcdP and/or HEV light (**a**) with no pretreatment or (**b**) following a 24 h pretreatment with 10 µM of α-tocopherol. Anti-tubulin α and anti-histone H3 antibodies were used to assess cytosolic and nuclear fraction purity, respectively. Ponceau S staining was used as a loading control. (**c**–**f**) Quantified results from three independent experiments (N = 3) indicating (**c**) AhR and (**d**) Nrf2 relative levels in cytosolic and nuclear extracts from IcdP and/or HEV-exposed cells; (**e**) AhR and (**f**) Nrf2 relative levels in cytosolic and nuclear extracts of cells treated with 10 µM of α-tocopherol prior to IcdP and/or HEV exposure. For each time point, AhR and Nrf2 signals were normalized to total protein staining and normalized signals in cytosolic fractions of control (C) cells were set as a baseline (value 1). Error bars are SEM; * *p* < 0.05, ** *p* < 0.01, *** *p* < 0.001 [One-way analysis of variance (ANOVA) with Dunnett’s procedure as post hoc test]. M = Protein molecular-weight markers, C = Control, I = IcdP, H = HEV, I + H = IcdP/HEV.

Accelerated ROS buildup and increased bulky DNA adducts suggest an impact of IcdP/HEV co-exposure on the metabolic response of cells to IcdP. We thus analyzed the activation of AhR and Nrf2, the main transcription factors regulating the genes involved in phases I and II of IcdP metabolism. As activation of both factors entails their nuclear translocation, we measured AhR and Nrf2 relative levels in the cytosol and nuclei of cells exposed to 500 nM of IcdP and/or 160 J/cm^2^ of HEV light, 0, 1, or 3 h post-exposure (Figure 3). Unexposed cells and cells exposed to either IcdP or HEV light alone have no detectable levels of AhR active form (red arrow) in their nuclei. However, active AhR is significantly present in the nuclei of IcdP/HEV-exposed cells immediately (0 h) at the end of co-exposure. AhR cytosolic and nuclear levels then recede at 1 h and are not detectable after 3 h in IcdP/HEV-exposed cells (Figure 3a,c). We only observed a significant presence of Nrf2 in the nuclei of IcdP/HEV-exposed cells 0 h after exposure, as evidenced by the fact that Nrf2 is more present in the nucleus than in the cytosol. However, at all time points, contrary to unexposed cells and cells exposed to either IcdP or HEV light alone, we found nearly undetectable levels of Nrf2 in the cytosol of IcdP/HEV-exposed cells (Figure 3a,d).

Interestingly, pre-treatment with 10 µM of α-tocopherol abolishes IcdP/HEV-induced nuclear translocation of AhR and prevents AhR cytosolic decline after the end of co-exposure (Figure 3b,e). α-Tocopherol pre-treatment also prevents IcdP/HEV-induced loss of the Nrf2 cytosolic pool. Moreover, it seems to stimulate Nrf2 nuclear translocation 3 h post-exposure in response to HEV light exposure and to IcdP/HEV co-exposure (although the increase in Nrf2 nuclear levels did not reach statistical significance—Figure 3b,f). In summary, IcdP/HEV co-exposure induces a transient sur-activation of AhR and is simultaneously associated with decreased levels of Nrf2. Both events are restricted in presence of α-tocopherol.

### 2.5. IcdP/HEV Co-Exposure Leads to Transcriptional Over-Activation of CYP1 Genes

**Figure 4:**
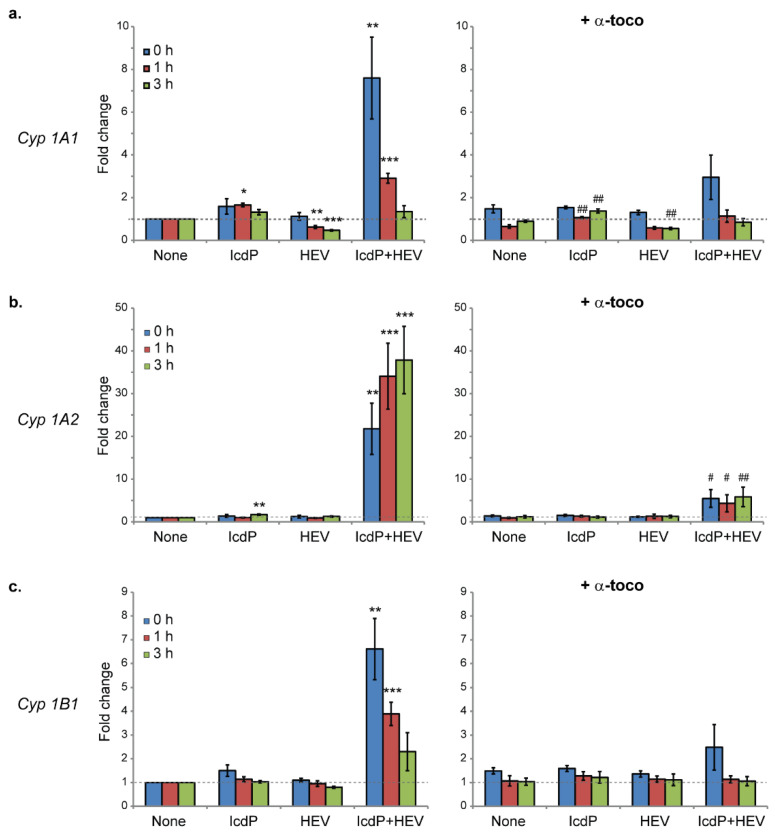
*CYP1* genes expression in RPE cells in response to IcdP and/or HEV light exposure. ARPE19 cells were exposed to IcdP (500 nM) and/or HEV light (160 J/cm^2^), with no pretreatment (lefts panels) or following a 24 h pretreatment with 10 µM of α-tocopherol (right panels). Relative mRNA levels for (**a**) 1A1, (**b**) 1A2, and (**c**) 1B1 were assessed by RT-qPCR, 0 h, 1 h, or 3 h following exposure. GAPDH housekeeping gene expression was used as an endogenous control at each time point. For each gene, normalized levels in the untreated/unexposed control were set as a baseline (value 1). At least four independent samples were analyzed for each condition investigated, and each sample was processed in quadruplicate (n = 4, N = 4). Error bars are SEM; #; * *p* < 0.05, ##; ** *p* < 0.01, *** *p* < 0.001 [one-way analysis of variance (ANOVA) with Dunnett’s procedure as post hoc test].

**Figure 5:**
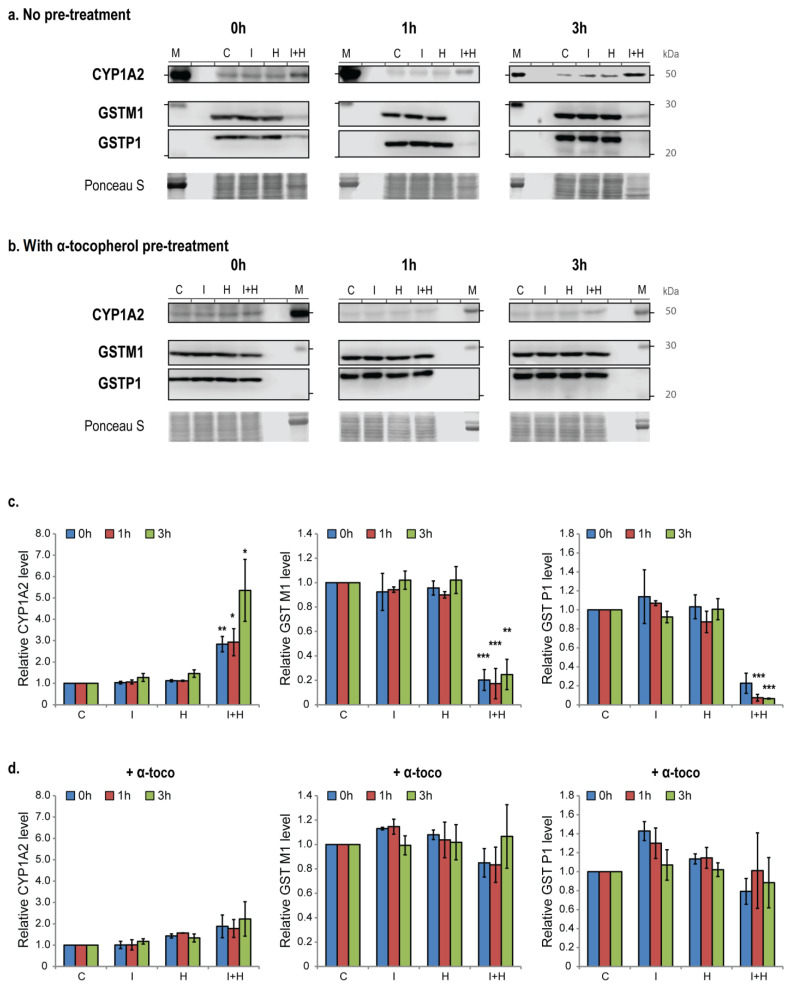
Changes in CYP and GST proteins levels in RPE cells following exposure to IcdP and/or HEV light. ARPE19 cells were exposed to IcdP (500 nM) and/or HEV light (160 J/cm^2^). Cells were lysed 0 h, 1 h, or 3 h following exposure. (**a**,**b**) Representative blots at 0, 1, and 3 h post-exposure for CYP1A2, GSTM1, and GSTP1 in cytosolic extracts from cells exposed to IcdP and/or HEV light (**a**) with no pretreatment or (**b**) following a 24 h pretreatment with 10 µM of α-tocopherol. Ponceau S staining was used as a loading control. (**c**,**d**) Quantified results from three independent experiments (N = 3) indicating CYP1A2, GSTM1 and GSTP1 relative levels at 0, 1, and 3 h post-exposure in IcdP and/or HEV-exposed cells (**c**) with no pretreatment or (**d**) following a 24 h pretreatment with 10 µM of α-tocopherol. For each time point, protein signals were normalized to total protein staining and normalized signals in control (C) cells were set as a baseline (value 1). Error bars are SEM; * *p* < 0.05, ** *p* < 0.01, *** *p* < 0.001 [One-way analysis of variance (ANOVA) with Dunnett’s procedure as post hoc test]. M = Protein molecular-weight markers, C = Control, I = IcdP, H = HEV, I + H = IcdP/HEV.

We sought to assess whether IcdP/HEV-induced changes in AhR and Nrf2 activation patterns translate into changes in the transcriptional regulation of genes encoding the enzymes responsible for IcdP metabolism. Using RT-qPCR, we first analyzed the time-dependent expression of phase I inducible CYP1 monooxygenases genes: *CYP1A1*, *1A2,* and *1B1*. Protein levels in the cytosol were then assessed using western blot analysis. mRNA (Figure 4) and protein (Figure 5) levels were measured 0, 1, and 3 h following an exposure to IcdP (500 nM) and/or HEV light (160 J/cm^2^).

Exposure to IcdP alone induces relatively small (1.7 fold) but statistically significant increases in *1A1* mRNA levels 1 h post exposure and in *1A2* mRNA 3 h post exposure (Figure 4a,b, lefts panels). HEV exposure alone significantly reduces *1A1* gene expression, but has no effect on *1A2* and *1B1*. By contrast, co-exposure to IcdP and HEV light causes a rapid and strong induction of all 3 *CYP1* genes expression. This induction is maximal immediately following co-exposure for *1A1* (7.6 fold) and *1B1* (6.6 fold), and recede with time to return to near-basal levels 3 h post-exposure (Figure 4a,c, left). Compared to *1A1* and *1B1*, a stronger IcdP/HEV-induced activation was observed for the *1A2* gene, with its 0 h post IcdP/HEV exposure mRNA level being 21.8 times its basal level (Figure 4b, left). In contrast to the other genes, *1A2* mRNA levels continue to increase during the recovery period, with the transcript level being 34.1 and 37.8 times its basal level at 1 h and 3 h post-exposure, respectively. Our data thus indicate a strong enhancement of *CYP1* genes induction triggered in RPE cells in response to IcdP and HEV light co-exposure.

CYP1A1 and CYP1B1 protein levels could not be detected in ARPE19 cells through western blot analysis (Figure S2). However, enhanced IcdP/HEV-induced *1A2* expression is associated with a higher content of CYP1A2 in the cytosol of co-exposed cells. At 0 and 1 h post-exposure, CYP1A2 is three times more abundant in the cytosol of co-exposed cells than in unexposed cells or cells exposed only to IcdP or HEV. At 3 h post-exposure, its level is 5.4 times higher (Figure 5a,c).

A 24 h pre-incubation with 10 µM of α-tocopherol significantly increases *1A1* and *1B1* mRNA basal levels in unexposed cells 1.5 times (Figure 4a–c, right panels). However, it drastically limits IcdP/HEV- induced activation of all three *CYP1* genes. In cells treated with α-tocopherol prior to co-exposure, *1A1* (Figure 4a, right) and *1B1* (Figure 4c, right) mRNA levels at 0 h post-exposure are increased to 2.9 and 2.5 times their basal expressions, respectively (*i.e.,* levels in untreated/unexposed cells). Moreover, basal levels are restored within 1 h post-exposure, for both genes. In the case of the *1A2* gene (Figure 4b, right), α-tocopherol pre-treatment limits the 0 h post-exposure induction to 5.5 times, and *1A2* transcript levels remain constant during the 3 h post-exposure period.

### 2.6. IcdP/HEV Co-Exposure Is Associated with a Depletion of GST Enzymes

**Figure 6:**
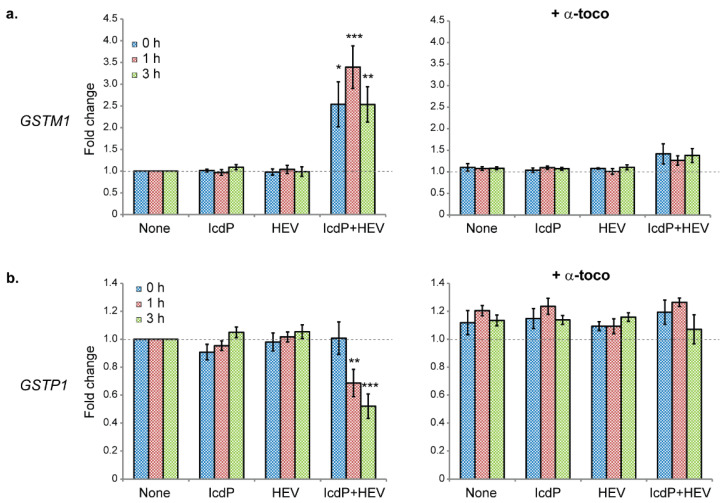
*GST* genes expression in RPE cells in response to IcdP and/or HEV light exposure. ARPE19 cells were exposed to IcdP (500 nM) and/or HEV light (160 J/cm^2^) with no pretreatment (lefts panels), or following a 24 h pretreatment with 10 µM of α-tocopherol (right panels). Relative mRNA levels for (**a**) GSTM1 and (**b**) GSTP1 were assessed 0 h, 1 h, or 3 h following exposure. At each time point, gene expression was normalized to GAPDH expression, and normalized expression levels in untreated/unexposed control were set as a baseline (value 1). At least four independent samples were analyzed for each condition investigated, and each sample was processed in quadruplicate (n = 4, N = 4). Error bars are SEM; * *p* < 0.05, ** *p* < 0.01, *** *p* < 0.001 [one-way analysis of variance (ANOVA) with Dunnett’s procedure as post hoc test].

Finally, we investigated the time-dependent expression of two phase II genes, *GSTM1* and *GSTP1*, as well as GSTM1 and GSTP1 cytosolic levels in RPE cells 0, 1, and 3 h following an exposure to IcdP (500 nM) and/or HEV light (160 J/cm^2^) (Figure 5 and Figure 6). *GSTM1* (Figure 6a, left panel) and *GSTP1* (Figure 6b, left) expressions are not affected by IcdP or HEV-light separated exposures. We found *GSTM1* gene expression to be upregulated after IcdP/HEV co-exposure, with maximal induction (3.4 fold the basal level) reached 1 h following co-exposure (Figure 6a, left). In contrast, *GSTP1* gene expression is not modulated immediately following co-exposure, but its expression is significantly down-regulated to 0.7 and 0.5 times the basal mRNA level after 1 h and 3 h, respectively (Figure 6b, left). Pre-treating RPE cells with α-tocopherol prevents both IcdP/HEV-related upregulation of *GSTM1* expression (Figure 6a, right) and post-exposure repression of the *GSTP1* gene (Figure 6b, right).

At the protein level, immediately at the end of exposure, we observed that the cytosolic content of both GSTM1 and GSTP1 is 80% lower in IcdP/HEV-exposed cells than in unexposed cells. GSTP1 cytosolic levels continue to decrease following exposure, and are 94% depleted after 3 h (Figure 5a,c). However, we detected no significant variations in GSTM1 and GSTP1 cytosolic levels following IcdP/HEV exposure, when cells were incubated with α-tocopherol prior to co-exposure (Figure 5b,d). With α-tocopherol pre-treatment, there is no significant difference between the CYP1A2 cytosolic level in IcdP/HEV-exposed cells and the level in unexposed cells (Figure 5b,d).

## 3. Discussion

Interactions between multiple risk factors have long been suspected to produce mechanisms underlying complex diseases like AMD. For AMD, one such interaction might involve xenobiotics from cigarette smoke and HEV light exposure. We have previously described that simultaneous exposure to IcdP and HEV light synergistically leads to important phototoxic stress and promotes AMD-like defects in human RPE cells [21], but the underlying mechanisms remain largely unknown. The present study thus aimed to gain mechanistic insight into the molecular pathways triggered by this toxic interaction.

### 3.1. Photo-Oxidative Stress Is Not the Driving Mechanism of IcdP/HEV Synergistic Toxicity

Benzo[a]pyrene (BaP), a major high-molecular-weight PAH found in cigarette smoke, has been reported to sensitize DNA to oxidative damage in the presence of ultraviolet-A (UVA) radiation by producing ROS through both type I and II photosensitized oxidation reactions [51,52,53]. Thus, we first addressed IcdP photosensitization properties under HEV light in RPE cells (Figure 1). Using a quencher of molecules in a triplet excited state or scavengers of ROS generated through the two processes, we investigated the involvement of photosensitized oxidation reactions mediated by HEV-excited IcdP in ROS synergistic formations and in IcdP/HEV-induced loss of RPE cells’ viability.

The reduction of ROS burden by both the superoxide anion (O_2_^•−^) dismutase mimetic agent TEMPOL (Figure 1c) and the singlet oxygen (^1^O_2_) quencher NaN_3_ (Figure 1d) indicates that multiple kinds of ROS are effectively produced following IcdP/HEV co-exposure. However, we found no or minor improvements in cell viability associated with the reduction of ROS (Figure 1c,d). Although the results with NaN_3_ suggest some occurrences of type II photosensitization process, quenching of triplet excited states by sorbate ions had no impact on cell viability or on ROS production in IcdP/HEV-exposed cells (Figure 1b). Taken together, these results strongly suggest that only a small portion of all IcdP/HEV-induced ROS is produced through photosensitized oxidation reactions.

In IcdP/HEV-exposed cells, we found an accelerated rate of ROS production sustained for an extended period after exposure, indicating activation of a long-lasting endogenous source of ROS, and/or strongly impaired antioxidant capacities of cells (Figure 2a,b). Strengthening antioxidant defenses with broad spectrum ROS scavengers like α-tocopherol or NAC efficiently prevents the oxidative stress induced by co-exposure (Figure 2a,b). However, we have shown that IcdP/HEV-induced cell viability loss could be prevented by α-tocopherol, but not by NAC [21]. Aside from its antioxidant properties, α-tocopherol has been reported for its ability to modulate phase I and II metabolic pathways [54,55]. Based on the data, oxidative stress is likely not responsible for IcdP/HEV synergistic toxicity, and appears as a deleterious consequence of a primary mechanism of toxicity triggered by the co-exposure.

### 3.2. IcdP/HEV Co-Exposure Induces an Unbalanced Metabolic Response in RPE Cells

The large amount of intracellular ROS caused by IcdP exposure under HEV light might result from PAH-inducible CYP1 monooxygenases of phase I metabolism [23,24]. Due to inefficient coupling of their catalytic cycle, ROS are released during CYP1 enzymatic action [24]. An increased catalytic cycle uncoupling caused by photo-excited IcdP could explain the increased ROS production in IcdP/HEV co-exposed cells. However, such production may also reflect a CYP1 activity that has been over-enhanced in response to co-exposure. We thus investigated the global effect of IcdP and HEV-simultaneous exposure on the induction of RPE cells’ metabolic response.

In addition to ROS production, another matter of concern related to CYP1 action is the conversion of PAH into genotoxic metabolites [25], like the well-documented benzo(a)pyrene 7,8-diol-9,10-epoxide derived from BaP. Without timely conjugation of such intermediates by phase II enzymes, they covalently bind to macromolecules including DNA, yielding bulky adducts [27,28,29,56,57].

Our results indicate that formation of DNA binding species does occur in IcdP/HEV-exposed cells (Figure 2c). Exposure of cells to 500 nM of IcdP alone produces undetectable levels of polymerase-blocking DNA lesions in our conditions, whereas the same amount of IcdP in the presence of HEV light leads to an accumulation of such lesions. Cell-free experiments confirm that IcdP biotransformation is required for formation of these lesions in the presence of HEV light (Sup material and methods and Figure S3a). We also ascertained that the events leading to bulky adducts formation relies on the absorption of light by IcdP and not by an endogenous chromophore (Figure S3b,c). Indeed, when cells are exposed to BaP, which only marginally absorbs in the HEV range [21], no accumulation of BaP-related bulky lesions is observed in DNA following HEV irradiation (Figure S3b). In contrast, when RPE cells are exposed to UVA in the presence of IcdP or BaP, which both absorb UVA wavelengths, an accumulation of bulky DNA adducts is observed for both compounds (Figure S3c). Accordingly, light-excited PAH triggers RPE cells’ metabolic response that seems to differ from the one elicited by unexcited PAH. Taken together, these results indicate that the signaling pathway controlling PAH metabolism is partly responsible for IcdP/HEV-induced synergic toxicity in RPE cells. In addition, the fact that α-tocopherol suppresses the accumulation of IcdP/HEV-induced bulky DNA adducts (Figure 2d) suggests that it activates compensatory mechanisms or interferes with the induction of RPE cells’ metabolic response to light-excited IcdP.

### 3.3. Unbalanced Activation of Phase I and II Metabolism after IcdP/HEV Co-Exposure

To confirm any IcdP/HEV-induced disruption in the signaling pathway responsible for IcdP metabolism, we analyzed the protein level and activation of AhR and Nrf2, the two key regulators of phase I and II gene expression.

We found that IcdP and HEV light co-exposure induces a strong but transient accumulation of AhR in RPE cells’ nuclei, followed by a massive reduction of AhR level in cells within 3 h after exposure (Figure 3a,c). This is reminiscent of the rapid degradation (within 4 h) of AhR observed only after ligand binding with strong and stable agonists and gene induction in a variety of cell types to control the level of activated AhR. As well as to attenuate AhR-mediated gene induction [58,59,60]. It thus supports the idea of a stronger activation of AhR in response to IcdP/HEV exposure of RPE cells. In accordance with this, we found that AhR’s strong presence in nuclei of IcdP/HEV-exposed cells coincide with substantially higher activation of all three *CYP1* genes’ transcription during co-exposure (Figure 4, left panels). Consistent with the gradual disappearance of AhR from cells within 3 h, and with the rapid turnover documented for *CYP1A1* and *1B1* messengers in mammal cells [61,62], *1A1* and *1B1* mRNA levels gradually decline after the co-exposure (Figure 4a,c). Both proteins’ levels were not detectable in our conditions in ARPE19 cells (Figure S2). In contrast, for *1A2*, the most markedly activated gene, the transcript level continues to increase following the end of IcdP/HEV co-exposure (Figure 4b), and a considerable accumulation of CYP1A2 proteins is observable 3 h following co-exposure (Figure 5a,c).

**Figure 7:**
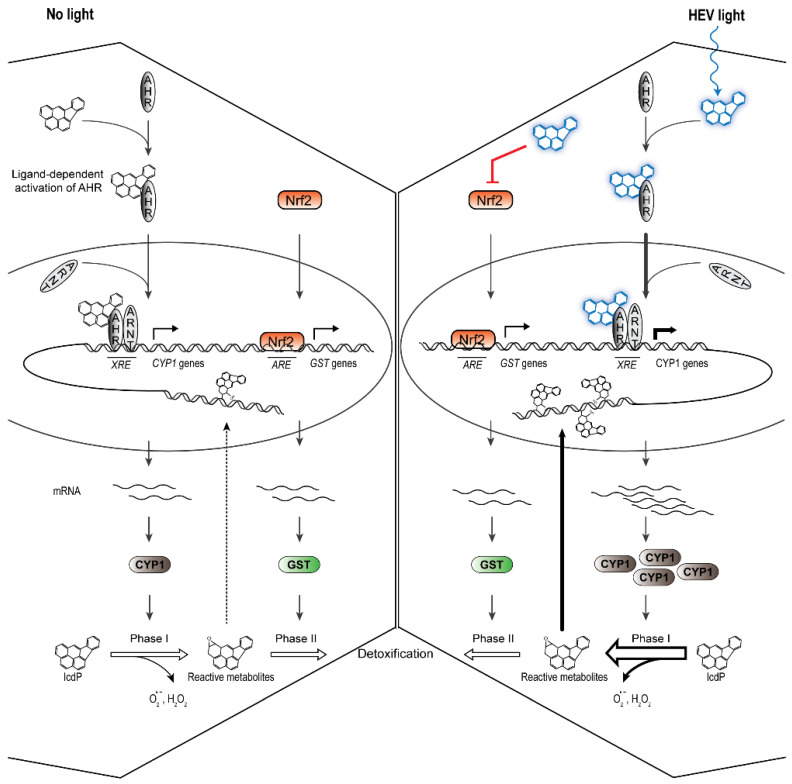
Theoretical model for differential IcdP-induced transcriptional regulation of PAH metabolism in the presence or absence of HEV light based on the presented results. In the absence of HEV light (no light, left panel): IcdP binding to AhR results in AhR release from a molecular chaperone complex and translocation to the nucleus. It then interacts with ARNT to form a transcriptionally active heterodimer. The activated complex binds to consensus regulatory sequences (XRE) located in the promoter region of target genes such as *CYP1* genes to induce their expression. CYP1 proteins catalyze the initial oxidation of IcdP, required for further IcdP processing, with a concomitant release of ROS. Biotransformation through oxidative phase I leads to the formation of IcdP metabolites, some of which are highly reactive. ROS and metabolites generated during phase I promote Nrf2 activation and nuclear translocation. This results in the binding of Nrf2 to ARE enhancer sequences that mediate the transcriptional activation of phase II target genes, including *GST* genes. GST enzymes conjugate IcdP metabolites with hydrophilic moieties to form inert excretable compounds. In the presence of HEV light (HEV light, right panel): binding of photo-excited IcdP to AhR promotes higher AhR activation and/or stabilization. This results in increased formation of active AhR/ARNT complexes and an over-enhanced induction of *CYP1* gene transcription. Simultaneously, light-excited IcdP promotes Nrf2 loss, resulting in cells’ inability to maintain an appropriate pool of functional GST proteins. Without timely conjugation by GST proteins, reactive IcdP intermediates are yielded at a rate exceeding the phase II capacity to eliminate them. These intermediates interact with macromolecules such as DNA, yielding covalent adducts. As a deleterious consequence, ROS accumulation and DNA damage occur.

Over-activation of AhR in response to IcdP/HEV light co-exposure is thus corroborated by the large presence of AhR in nuclei by *CYP1* gene upregulation and by CYP1A2 protein accumulation. This—along with increased rates of ROS buildup and bulky DNA adduct accumulation 6 h following co-exposure (Figure 2)—supports the hypothesis of a light-excited IcdP-induced over-enhancement of phase I metabolism. The reasons for such an enhancement remain unknown. We propose that it results from an increased binding affinity of HEV-excited IcdP for AhR. Photo-excited IcdP may thus improve the nuclear translocation of AhR, thereby enhancing the formation of active transcription complexes (Figure 7, right). It should be mentioned that some PAH metabolites have been found to be more potent inducers of *CYP1* gene expression than their parent PAH [63]. In rat skin, IcdP metabolites are detected as early as 30 min after topical application of IcdP to the skin [29]. Therefore, it cannot be ruled out that phase I enhancement in RPE cells is mediated by a light absorbing metabolite of IcdP.

Simultaneously with AhR over-activation, IcdP/HEV co-exposure is associated with a considerable loss of Nrf2, the main regulator of phase II gene induction (Figure 3a,d). Nrf2 is also critical for regulating ROS accumulation and cell response to oxidative stress. The observed loss of Nrf2 may significantly contribute to accelerating ROS buildup measured in IcdP/HEV co-exposed cells. Correlating with the Nrf2 disappearance and weak nuclear presence, *GSTP1* gene expression is strongly downregulated following IcdP/HEV co-exposure (Figure 6b, left). However, *GSTM1* appears transiently upregulated, albeit to a lesser extent than *CYP1* genes (Figure 6a, left). Strikingly, both proteins’ levels drop substantially in IcdP/HEV-exposed cells, suggesting an IcdP/HEV-induced degradation of these proteins (Figure 5a,c). Our results strongly indicate that Nrf2 disappearance is associated with cells’ inability to maintain an appropriate pool of functional GST proteins after co-exposure.

Considering ROS buildup and the higher IcdP-related DNA adduct accumulation in co-exposed cells, IcdP/HEV-induced loss of Nrf2, GSTM1, and GSTP1 proteins supports the idea that phase II metabolism is hindered in response to co-exposure. As a consequence, phase II may not be sufficient to sustain the enhanced CYP1 induction. Phase I and II thus appear uncoupled, with an over-activated phase I yielding reactive metabolites at a rate exceeding the phase II capacity to conjugate them (Figure 7).

### 3.4. α-Tocopherol Prevents Phase I and II Uncoupling

In view of α-tocopherol’s capacity to prevent ROS and DNA damage accumulation in IcdP/HEV-exposed RPE cells (Figure 2a,d), we analyzed how it impacts AhR- and Nrf2-mediated regulation pathways in IcdP/HEV-exposed cells. In the presence of α-tocopherol, no nuclear translocation of AhR could be detected immediately following IcdP/HEV exposure. Moreover, AhR cytosolic level is slightly increased following co-exposure, and ligand-binding-induced massive degradation of AhR is not observed anymore (Figure 3b,e). It strongly suggests that binding of AhR with a strong stable agonist and AhR activation are much less major events in IcdP/HEV-exposed cells in the presence of α-tocopherol. Suppression of IcdP/HEV-induced AhR nuclear translocation in the presence of α-tocopherol is associated with a strong attenuation of *CYP1* genes’ over-activation (Figure 4, right panels) and with CYP1A2 proteins’ accumulation prevention (Figure 5b,d). α-Tocopherol has previously been reported to be a weak agonist of AhR, as well as to decrease PAH-induced CYP1 activity in vivo [54,55]. Although this is in agreement with our findings, it was suggested that α-tocopherol’s inhibitory effect is produced at the post-translational level. However, our data indicate that α-tocopherol can also limit the signal transduction induced by the interaction between IcdP and HEV light, which is responsible for phase I gene over-expression. α-tocopherol may act by directly quenching the photo-excited molecule. However, since α-tocopherol can bind AhR, its action may result from a restricted availability of binding sites involved in light-excited IcdP binding to AhR.

Interestingly, α-tocopherol also offsets IcdP/HEV-induced disruption of phase II regulation. In the presence of α-tocopherol, the cytosolic pool of Nrf2 is largely preserved following IcdP/HEV exposure (Figure 3b,f). In addition, Nrf2 tends to be delivered to cell nuclei 3 h after co-exposure, showing that cells are still subject to significant stress. Nrf2 preservation and activation are associated with a complete restoration of *GSTM1* and *GSTP1* expression levels (Figure 6, left), and with ongoing maintenance of their protein levels (Figure 5b,d). α-Tocopherol thus seems able to reduce IcdP/HEV impact on both phases, ensuring that they remain balanced.

## 4. Materials and Methods

All experiments were conducted in accordance with our institution’s guidelines and the Declaration of Helsinki.

### 4.1. Cell Culture

All experiments were performed on confluent monolayers of spontaneously immortalized human RPE cell line ARPE19 (ATCC^®^ CRL-2302™). ARPE19 cells were cultured in high-glucose Dulbecco’s Modified Eagle’s Medium (DMEM) (Corning Cellgro, Manassas, VA, USA), supplemented with 10% fetal bovine serum (Wisent Inc., Saint-Jean-Baptiste, QC, Canada), 100 U.I./mL penicillin, and 100 µg/mL streptomycin (Wisent Inc.) at 37 °C and 5% CO_2_. Confluent monolayers were allowed to stabilize at least 2 weeks before use.

### 4.2. Light Source and Dosimetry

Cell monolayers were exposed to a light spectrum comparable to the solar spectrum reaching the adult human retina, as we previously described [21]. The light source consisted of an Oriel 1.6 kW solar simulator (SSL) combined with an air mass 1.5 G filter (Newport Corporation, Irvine, CA, USA) and with a yellow GG420 long pass optical filter (Schott, Lebanon, PA, USA). The output was measured prior to each irradiation using a 1918-R Optical Power Meter (with an 818 P high-power thermopile detector; Newport Corporation), and the fraction of the output in the HEV light range (400–500 nm) was determined according to the manufacturer’s instructions. HEV irradiance on the cell surface was ~22 mW/cm^2^.

### 4.3. Indeno[1,2,3-cd]Pyrene Exposure and Light Irradiation Procedure

Indeno[1,2,3-cd]pyrene (IcdP; Cerilliant^®^ analytical standard, Sigma-Aldrich, Oakville, ON, Canada) was dissolved in dimethyl sulfoxide (DMSO). ARPE19 cells were incubated with the indicated concentrations of IcdP, or with the vehicle (DMSO) in phosphate buffered saline (PBS—with calcium and magnesium) for 30 min at 37 °C in the dark. Cells were then exposed to 160 J/cm^2^ of HEV light in a cooling box (~120 min of irradiation), a dose equivalent to that received in 4 h of direct sunlight exposure. Unirradiated samples were kept in the dark at 4 °C throughout the duration of irradiation. IcdP or vehicle solutions were removed immediately at the end of irradiation. Cells were then harvested with trypsin/EDTA (Thermo Fisher Scientific, Mississauga, ON, Canada), or allowed to recover in complete DMEM at 37 °C until analysis.

### 4.4. Antioxidant/Quencher Treatments

As a preliminary step before use, the effect of increasing concentrations of each antioxidant/quencher (all from Sigma Aldrich, Oakville, ON, Canada) on ARPE19 cells’ metabolic activity was initially assessed using the colorimetric MTS assay for viable cell metabolic activity (assay detailed in “Cytotoxicity assessment”). Concentrations selected for the present study produced no significant variation of metabolic activity over 48 h. Cells were thus incubated with 1 µg/mL of ethyl sorbate (EthS), 1.5 mM of 4-hydroxy-1-oxyl-2,2,6,6-tetramethylpiperidine (TEMPOL), 10 mM of sodium azide (NaN_3_), 10 μM of α-tocopherol (α-toco), or 5 mM of *N*-acetylcysteine (NAC) in complete DMEM for 18–24 h prior to IcdP and/or HEV exposure. The agent was added back to PBS during exposure and to culture media post-exposure.

### 4.5. Cytotoxicity Assessment

The cytotoxicity of IcdP and/or HEV light exposure (in the presence or absence of antioxidant/quencher) was assessed 24 h post-exposure using a colorimetric MTS assay for viable cell metabolic activity (CellTiter 96^®^ AQueous Non-Radioactive Cell Proliferation Assay; Promega, Fitchburg, WI, USA) according to the manufacturer’s protocol. Briefly, MTS assay reagents were added to the cell monolayer medium. Plates (96-well) were incubated for 2 h at 37 °C, and optical densities were read at 490 nm using a microplate reader (Biorad 550 Microplate reader; Biorad, Hercules, CA, USA). For each treatment/exposure, after correcting for background absorbance, the average absorbance of DMSO-treated controls was used as a baseline metabolic activity (value 1). In each experiment, the conditions investigated were assayed in quadruplicate (n = 4), and experiments were independently repeated at least three times (N ≥ 3).

### 4.6. ROS Content and Accumulation Kinetics

Intracellular ROS content was measured using the oxidant-sensitive fluorescent probe chloromethyl-2’,7’-dichlorodihydrofluorescein diacetate, acetyl ester (CM-H_2_DCFDA; Thermo-Fisher, Mississauga, ON, Canada), and assayed as previously described [64] with minor modifications [21]. Immediately at the end of IcdP and/or HEV exposure (−/+ antioxidant/quencher), cells were incubated with 5 µM CM-H_2_DCFDA in PBS (−/+ antioxidant/quencher) for 30 min at 37 °C. CM-H_2_DCFDA was replaced by PBS (−/+ antioxidant/quencher), and cells were further incubated at 37 °C for up to 6 h. The fluorescence of cells in each well was recorded at different time points following the CM-H_2_DCFDA addition as we previously described [21].

To assess ROS accumulation after exposure, data were taken 0 and 60 min following CM-H_2_DCFDA addition. A percentage increase in intracellular levels of ROS was determined at 60 min, as described by Wang and Joseph [64]. Each condition investigated was assayed in quadruplicate (n = 4), and experiments were independently repeated at least three times (N ≥ 3). To derive kinetics of ROS accumulation, data were taken 0, 30 min, and every hour up to 6 h following CM-H_2_DCFDA addition. A percentage increase in the intracellular levels of ROS was determined at each time point. The average increase at 30 min of unirradiated DMSO-treated controls was used as a baseline (value 1). Each condition investigated was independently tested four times (N = 4).

### 4.7. IcdP-Related Genotoxicity Assessment

Stable bulky adducts derived from the covalent modification of DNA by PAH metabolites have been shown to strongly block replication by DNA polymerases [65,66,67]. The formation of bulky DNA adducts derived from IcdP in the presence or absence of HEV light exposure (−/+ antioxidant) was thus assessed 0 and 6 h post-exposure using a modified large amplicon quantitative PCR assay (LA-QPCR) [68,69]. Quantification of adducts by LA-QPCR is based on the decreased yield of damaged template amplification due to the bulky-lesions-induced blockage of DNA polymerase [70]. LA-QPCR was performed according to a protocol modified from Furda et al. to allow for simultaneous amplification of large and short targets under the same PCR parameters [71]. Primers for large (4889 bp) and short (66 bp) nuclear targets were designed for the human corin, serine peptidase gene (*CORIN*), using the primer designing tool Primer-BLAST [72]. Primers sequences are depicted in Table S1.

Briefly, ARPE19 cells were harvested 0 h or 6 h after IcdP and/or HEV light exposure. DNA was extracted with the DNeasy Blood and Tissue Kit (Qiagen, Toronto, ON, Canada) according to the manufacturer’s protocol, with an RNase treatment. Amplification was achieved using a Rotor-Gene Q cycler (Qiagen). Reactions consisted of 20 ng of total DNA, 0.5 units of Deep Vent (exo -) polymerase (New England Biolabs, Ipswich, MA, USA) with 1× supplied ThermoPol^®^ Reaction Buffer, 0.2 µM of each primer, 250 µM of each dNTP (Thermo Fisher Scientific, Mississauga, ON, Canada), and 1× Evagreen^®^ dye (Biotium Inc., Fremont, CA, USA) in a final reaction volume of 20 µL. PCR cycles were 95 °C for 10 min, followed by 40 cycles of 95 °C for 45 s, 64 °C for 50 s, and 72 °C for 210 s. The same calibrator (DNA from untreated ARPE19 cells) and a negative control (no DNA) were included in each run. For each sample, a relative frequency of blocking damage in DNA was defined as an “amplification delay between large and short targets of sample”/“amplification delay between large and short targets of calibrator” ratio, and calculated using the 2^ΔΔCt^ method, where ΔΔCt = ΔCt_sample_ − ΔCt_calibrator_ = (Ct^4889^ − Ct^66^)_sample_ − (Ct^4889^ − Ct^66^)_calibrator_. At least three independent samples were analyzed for each condition investigated (N ≥ 3), and each sample was processed in quadruplicate (n = 4).

### 4.8. Expression of Genes Involved in PAH Metabolism

Gene expression was quantified through real-time quantitative PCR (RT-qPCR) in cells exposed to IcdP and/or HEV (−/+ α-tocopherol pretreatment). Analyses were performed on genes coding for phase I (*CYP1A1*, *CYP1A2* and *CYP1B1*) and phase II (*GSTM1* and *GSTP1*) PAH metabolism enzymes. The *GAPDH* (glyceraldehyde 3-phosphate dehydrogenase) gene was used as a control. Sequences of specific primers for *CYP1A1*, *GSTM1,* and *GAPDH* were obtained from [39,73,74], respectively. Specific primers for other genes were designed using Primer-BLAST based on their Refseq accession number (Table S1).

Total RNA was extracted 0, 1, and 3 h following exposure using an RNA isolation kit (RNeasy Mini Kit from Qiagen, Toronto, ON, Canada) according to the manufacturer’s protocol. Reverse transcription of mRNA was performed on 750 ng of total RNA using the TaqMan Reverse Transcription Reagents (Thermo Fisher Scientific, Mississauga, ON, Canada) with oligo (dT)_16_ primers, according to the manufacturer’s recommendations. Quantitative PCR was achieved on 20 ng of cDNA. Reaction mixes included 1× Brilliant III Ultra-Fast SYBR^®^ Green qPCR Master Mix (Agilent Technologies, Saint-Laurent, QC, Canada) and 0.4 µM of each primer. PCR cycles were 95 °C for 10 min, followed by 40 cycles of 95 °C for 30 s and 60 °C for 45 s. The expression of each gene was normalized to *GAPDH* expression using the ΔCt method. Normalized levels of expressions in the control (DMSO-treated samples) were set as a baseline (value 1). At least four independent samples were analyzed for each condition investigated (N ≥ 4), and each sample was processed in quadruplicate (n = 4).

### 4.9. Protein Isolation and Western Blot Assays

Cytosolic and nuclear protein levels were analyzed using western blot analysis 0, 1, and 3 h following exposure of cells to IcdP and/or HEV (−/+ α-tocopherol pretreatment). For cytosolic and nuclear fractionation, cells were lysed 30 min on ice in 2 volumes of cytosolic lysis buffer (10 mM Tris-HCl pH 8.0; 340 mM sucrose; 3 mM CaCl_2_; 2 mM MgCl_2_; 0.1 mM EDTA; 1 mM DTT; 0.5% NP40 and 1× of complete, EDTA-free protease inhibitor cocktail (Roche, Mississauga, ON, Canada)) and centrifuged for 10 min at 3000× *g* and 4 °C. The cytosolic fraction (supernatants) was collected, assayed for protein concentration using the Pierce BCA Protein Assay Kit (Thermo Fisher Scientific, Mississauga, ON, Canada) according to the manufacturer’s protocol and stored at −20 °C until analysis. Pellets were washed with 1 volume of cytosolic lysis buffer, spun for 3 min at 3000 g and 4 °C, and resuspended in 2 volumes of high salt nuclear lysis buffer (20 mM HEPES pH 7.9; 1.5 mM MgCl_2_; 1 mM EDTA; 400 mM NaCl; 50 mM KCl; 1 mM DTT; 0.2% NP40; 10% glycerol and 1× cOmplete, EDTA-free protease inhibitor cocktail). Nuclei were broken on ice using pellet pestles for 1.5 microcentrifuge tubes, kept for 30 min on ice, and centrifuged 30 min at 22,000× *g* and 4 °C. The nuclear fraction (supernatants) was collected and stored at −20 °C until analysis.

AhR and Nrf2 translocation: cytosolic (40 µg of protein) and nuclear (1.5 × cytosolic vol) fractions were subjected to SDS-PAGE Western blot using primary rabbit polyclonal anti-AhR (Novus Biological, Oakville, ON, Canada; diluted 1:500) or goat polyclonal anti-human/mouse Nrf2 (R&D Systems, Minneapolis, MN, USA; diluted 1:250) antibodies. To assess the fractions’ purity, we used mouse anti-α-tubulin, clone DM1A (Abcam, Cambridge, UK; diluted 1:10,000), and rabbit polyclonal anti-histone H3 antibodies (Abcam; diluted 1:1000). Horseradish peroxidase (HRP) conjugated goat anti-rabbit IgG (Sigma-Aldrich, Oakville, ON, Canada; diluted 1:5000), donkey anti-goat IgG (Jackson ImmunoResearch Laboratories; diluted 1:5000), or goat anti-mouse IgG (Jackson ImmunoResearch Laboratories, PA, USA; diluted 1:1000) antibodies were used for secondary antibody incubations. Additional information on antibodies is provided in Table S2. Visualization was performed using enhanced chemiluminescent (ECL) detection, using the SuperSignal West Pico PLUS Chemiluminescent HRP Substrate (Thermo Fisher Scientific). Blots were imaged with a C-DiGit Blot Scanner (LI-COR Biosciences, Lincoln, NE, USA) and analyzed with Image Studio Lite software version 5.0 (LI-COR Biosciences; https://www.licor.com/bio/image-studio-lite/). Ponceau S staining was used as a loading control, and AhR or Nrf2 band intensities were normalized to ponceau S total protein staining intensity. Normalized signals in cytosolic fractions of control cells were set as a baseline (value 1). Experiments were independently repeated three times (N = 3).

*CYP1 and GST levels*: cytosolic proteins (40 µg) were subjected to SDS-PAGE Western blot using primary mouse anti-CYP1A2, clone D-3 (Santa Cruz Biotechnology, Dallas, TX, USA, diluted 1:100), mouse anti-GSTM1, clone 1H4F2 (Santa Cruz Biotechnology, diluted 1:200), or mouse anti-GSTP1, clone 3F2C2 (Santa Cruz Biotechnology, diluted 1:200) antibodies (Table S2). Secondary antibody incubation was performed with the HRP-conjugated goat anti-mouse IgG (Jackson ImmunoResearch Laboratories; diluted 1:1000) antibody, and blots were visualized and analyzed as described before. Ponceau S staining was used as a loading control, and for total protein normalization. Normalized signals in control cells were set as a baseline (value 1). Experiments were independently repeated three times (N = 3).

### 4.10. Statistical Analysis

Data are presented as mean ± standard deviation (SD) or standard error of mean (SEM). Statistical analysis was performed using GraphPad Prism 8 software (GraphPad Software, San Diego, CA, USA). For multi-group comparisons, analysis of variance (ANOVA) followed by Dunnett or Tukey HSD procedures as post hoc tests were used. Differences between two groups were assessed with the two-tailed homoscedastic student’s *t* test. Kinetic curves for ROS accumulation over time were compared with nonlinear regression models using the F-test. Significance level was defined for *p* value ≤ 0.05.

## 5. Concluding Remarks

In this work, we have found that absorption of HEV light by IcdP in RPE cells results in the over-induction of AhR signaling-dependent transcription of *CYP1* genes, in the loss of Nrf2-regulated maintenance of GST proteins, and in the loss of phase I and II metabolism coupling. This leads to bulky DNA damage and oxidative stress, and seems related to IcdP and HEV light synergic toxicity. Aside from *CYP1*, AhR regulates several genes and pathways whose disruption is detrimental to cells [75,76]. Moreover, the loss of Nrf2 severely limits cells’ capacity to cope with xenobiotic and oxidative stress.

Investigating the effects of co-exposure to HEV light and PAH is highly relevant for understanding the risk of complex diseases such as AMD. *GSTM1* mRNA levels are strongly reduced in AMD RPE [77], and reduced levels of GSTP1 have been found in AMD retinas [78], suggesting a relationship between specific GST isoforms and AMD pathogenesis. We found that IcdP and HEV co-exposure lowers *GSTP1* expression and promotes a depletion of more than 80% of GSTM1 and GSTP1. Bhosale et al. highlighted the important contribution of GSTP1 to the highly selective uptake, delivery, and accumulation within the central macula of zeaxanthin [79], a carotenoid with protective effects against AMD progression [80]. If sustained over time, the repression of *GSTP1* and GSTP1 depletion promoted by IcdP/HEV co-exposure may presumably explain, at least in part, the lower levels of macular carotenoids associated with tobacco use [81,82].

Aside from tobacco smoke, significant PAH concentration exposure can also result from ambient air polluted with waste incineration, vehicle exhaust, wood combustion, or fossil fuel pyrolysis in industrial and residential uses [83]. Noteworthily, increased risks of an altered RPE layer and AMD have recently been associated with pollution and higher concentrations of ultrafine particulate matter with an aerodynamic diameter of 2.5 µm or less in ambient air [84,85]. PM_2.5_, which mainly arises from combustion processes, include PAH. There are little data related to pollution risks with HEV-light exposure. Simultaneous exposure of RPE cells to PAH and HEV light is inevitable amongst smokers and people in polluted environments. This toxic interaction for RPE cells could explain the greater risk of people exposed to PAH developing AMD. In addition to highlighting the importance of protecting our retina against these toxic agents, the mechanistic understanding of this toxic synergy represents potential avenues to prevent or treat AMD.

## Data Availability

Data is contained within the article and supplementary materials.

## Supplementary Materials

The following supporting information can be downloaded at: https://www.mdpi.com/article/10.3390/ijms242417385/s1.
