# Supplementary material for: Indenopyrene and Blue-Light Co-Exposure Impairs the Tightly Controlled Activation of Xenobiotic Metabolism in Retinal Pigment Epithelial Cells: A Mechanism for Synergistic Toxicity
Source: Int J Mol Sci. 2023 Dec 12;24(24):17385. doi: 10.3390/ijms242417385 (PMC10744144; doi:10.3390/ijms242417385)
Supplement: Supplementary file 1 [file ijms-24-17385-s001.zip › ijms-2712479-supplementary.pdf]

Supplementary material

Indenopyrene and blue-light co-exposure impairs the tightly controlled activation of xenobiotic metabolism in retinal pigment epithelial cells: a mechanism for synergistic toxicity

Corinne Zinflou [a,b] and Patrick J. Rochette [a,b,c]

a. Axe Médecine Régénératrice, Centre de Recherche du CHU de Québec – Université Laval, Hôpital du Saint-Sacrement, Québec, Qc, Canada

b. Centre de Recherche en Organogénèse Expérimentale de l'Université Laval/LOEX, Université Laval, Québec, Qc, Canada

c. Département d'Ophtalmologie et ORL - chirurgie cervico-faciale, Université Laval, Québec, Qc, Canada

* Corresponding author at: Axe Médecine Régénératrice, Centre de Recherche du CHU de Québec – Université Laval, Hôpital du Saint-Sacrement, Québec, QC, Canada.
E-mail address: Patrick-J.Rochette@crchudequebec.ulaval.ca

Keywords: High-energy visible light (HEV; blue light), polycyclic aromatic hydrocarbons metabolism, indenopyrene (IcdP) phototoxicity, oxidative stress, retinal pigment epithelial (RPE) cells, aryl hydrocarbon receptor (AhR), nuclear factor erythroid-2 related factor-2 (Nrf2).

SUPPLEMENTARY MATERIALS AND METHODS

Detailed western blot procedure

Cytosolic (40 µg of protein) and nuclear (1.5 x cytosolic vol) fractions of each sample were denatured for 5 min at 95°C in 1X loading buffer (62.5 mM Tris-HCl pH 6.8, 10% glycerol, 2% SDS, 0.01% bromophenol blue, 2.5% β-mercaptoethanol) and separated by sodium dodecyl sulphate – polyacrylamide gel electrophoresis (SDS-PAGE) on a 10% (for AhR and Nrf2 translocation analyses) or a 12% resolving gel (for CYP1 and GST), for 45 min at 150 V. Proteins were then transferred to a 0.45 µm nitrocellulose membrane (Biorad, ON, CA) in 1X transfer buffer (25 mM Tris, 192 mM glycine, 10% (v/v) methanol) for 90 min at 100 V.

PAH treatment and/or UVA-visible exposure

UVA-visible exposure consists of exposure to a light spectrum comparable to the solar spectrum reaching the earth surface, as previously described [21]. Briefly, UVA-visible irradiation was performed using an Oriel 1.6 kW solar simulator (SSL) system to which a CGA-345 long pass filter (Schott, PA, USA) was added. UVA output was measured prior to each irradiation session using a UVX Radiometer (UVP Inc., CA, USA) with an UVA probe. UVA irradiance at cell surface was 2.5 mW/cm^2.

Confluent ARPE19 cells were incubated with the indicated concentrations of benzo[a]pyrene (BaP; Sigma-Aldrich, ON, CA), indeno[1,2,3-cd]pyrene (IcdP) or with vehicle (DMSO) in PBS (supplemented with calcium and magnesium) for 30 min at 37°C, 5% CO_2 in the dark. They were then exposed to 5 J/cm^2 of UVA-visible light in a cooling box using the SSL/CGA-345 setup (~33 min of irradiation). The amount of HEV light (determined according to the manufacturer instructions) transmitted to the cells along with UVA, corresponded to 60 J/cm^2. Unirradiated samples were kept in the dark at 4°C all along the irradiation. PAH or vehicle solutions were removed immediately at the end. Cells were allowed to recover in complete DMEM at 37°C for 6 h and then harvested with trypsin/EDTA. The relative frequency of polymerase-blocking lesions in DNA from PAH and/or UVA-visible light – exposed cells was estimated 6 h post-exposure by LA-QPCR, as described in "Materials and methods".

Cell-free experiments

Genomic DNA was extracted from untreated/unexposed ARPE19 cells with the DNeasy Blood and Tissue Kit (Qiagen, ON, CA) according to the manufacturer protocol, with an RNase treatment. DNA samples were quantified using a NanoDrop 2000 spectrophotometer (Thermo Fisher Scientific, ON, CA). 20 ng/µl of naked DNA in Rnase-Dnase free water was mixed with IcdP at a final concentration of 500 nM, and then exposed to 160 J/cm^2 of HEV light in a cooling box using the SSL/GG420 setup (see "Materials and methods"). Unirradiated DNA samples were kept in the dark at 4°C throughout the duration of irradiation. IcdP and/or HEV light exposure - induced formation of polymerase-blocking lesions in naked DNA was assessed, as described in "Materials and methods".

293T cell line culture and whole cell extract preparation for western blot

The human embryonic kidney 293T cell line (ATCC® CRL-3216™) was cultured to near-confluence (90%) in high glucose Dulbecco's Modified Eagle's Medium (DMEM) (Corning cellgro, VA, USA), supplemented with 10% fetal bovine serum (Wisent Inc, QC, CA), 100 U.I./ml penicillin and 100 µg/ml streptomycin (Wisent Inc) at 37°C, 5% CO_2. ARPE19 cells were handled as described in "Materials and methods". 293T or ARPE19 cells were harvested using trypsin/EDTA, lysed 15 min on ice in 500 µl of radio-immunoprecipitation assay (RIPA) buffer (50 mM Tris-HCl, pH 8.0; 150 mM NaCl; 1% NP40; 0.5% sodium deoxycholate; 0.1% SDS and 1X of cOmplete, EDTA-free protease inhibitor cocktail (Roche, ON, CA)) and centrifuged 15 min at 13000 g, 4°C for total protein

extraction. Whole cell extracts were assayed for proteins concentration using Pierce BCA Protein Assay Kit (Thermo Fisher Scientific, ON, CA).

40 µg of total protein from 293T or ARPE19 cells were subjected to SDS-PAGE Western blot as described above and in "Materials and methods", using primary mouse anti-CYP1A1 (clone B-4), mouse anti-CYP1B1 (clone G-4) or mouse anti-CYP1A2 (clone D-3) antibodies (Santa Cruz Biotechnology, TX, USA; diluted 1:100). Membranes were also probed with a goat polyclonal anti-β-actin antibody (Santa Cruz Biotechnology; diluted 1:2000). Secondary antibody incubation was performed with HRP-conjugated goat anti-mouse IgG antibody or donkey anti-goat IgG antibodies (Jackson ImmunoResearch Laboratories, PA, USA; diluted 1:5000). Ponceau S staining was used as a loading control.

SUPPLEMENTARY TABLES

Table S1: Primers used in PCR-based assays

Target	RefSeq accession #	Forward primer (5'→ 3')	Reverse primer (5'→ 3')	Product size (bp)
LA-QPCR				
CORIN large target	NG_032679	CTGTGAGGGCTCATAACCCA	CAGGAATTGGATCCCGTGGT	4889
CORIN short target		TGCAGCGCACCAGCATGG	AGGGTACATGTGCACATTGTGCAGGTTA	66
qPCR				
CYP1A1	NM_000499.5	AGTGGCAGATCAACCATGACCAGA	CCGCTTGCCCATGCCAAAGATAAT	134
CYP1A2	NM_000761.5	TCAGCCTCGTGAAGAACACTC	CCACAGGAACCTCTGGTTGA	137
CYP1B1	NM_000104.3	CTCAACGCTGTGAGGAAACC	GAGTCTCTTGGCGTCGTCAG	127
GSTM1	NM_000561.3	GAACTCCCTGAAAAGCTAAAGC	GTTGGGCTCAAATATACGGTGG	136
GSTP1	NM_000852.3	GGAGACCTCACCCTGTACCAG	ACATAGTCATCCTTGCCCGC	185
GAPDH	NM_002046.7	AAGGTCGGAGTCAACGGAT	GGAAGATGGTGATGGGATTTC	220

Table S2: Antibodies for western blot analysis

Antibody	Clone	Initial concentration (mg/ml)	Supplier	Catalogue #
Rabbit anti-AhR	polyclonal	1.0	Novus Biological	NB100-2289
Goat anti-human/mouse Nrf2	polyclonal	0.2	R&D Systems	AF3925
Mouse anti-α-tubulin	DM1A	1.0	Abcam	ab7291
Rabbit anti-histone H3	polyclonal	1.0	Abcam	ab1791
Mouse anti-CYP1A1	B-4	0.2	Santa-Cruz Biotechnology	sc-25304
Mouse anti-CYP1A2	D-3	0.2	Santa-Cruz Biotechnology	sc-393783
Mouse anti-CYP1B1	G-4	0.2	Santa-Cruz Biotechnology	sc-374228
Mouse anti-GSTM1	1H4F2	0.2	Santa-Cruz Biotechnology	sc-517262
Mouse anti-GSTP1	3F2C2	0.2	Santa-Cruz Biotechnology	sc-66000
Goat anti-β-actin	polyclonal	0.1	Santa-Cruz Biotechnology	sc-1616
HRP-conjugated goat anti-rabbit IgG	polyclonal	4.0 – 11.0	Sigma-Aldrich	A0545
HRP-conjugated donkey anti-goat IgG	polyclonal	-	Jackson ImmunoResearch	705-035-003
HRP-conjugated goat anti-mouse IgG	polyclonal	-	Jackson ImmunoResearch	115-035-003

* HRP = Horseradish peroxidase

SUPPLEMENTARY FIGURES

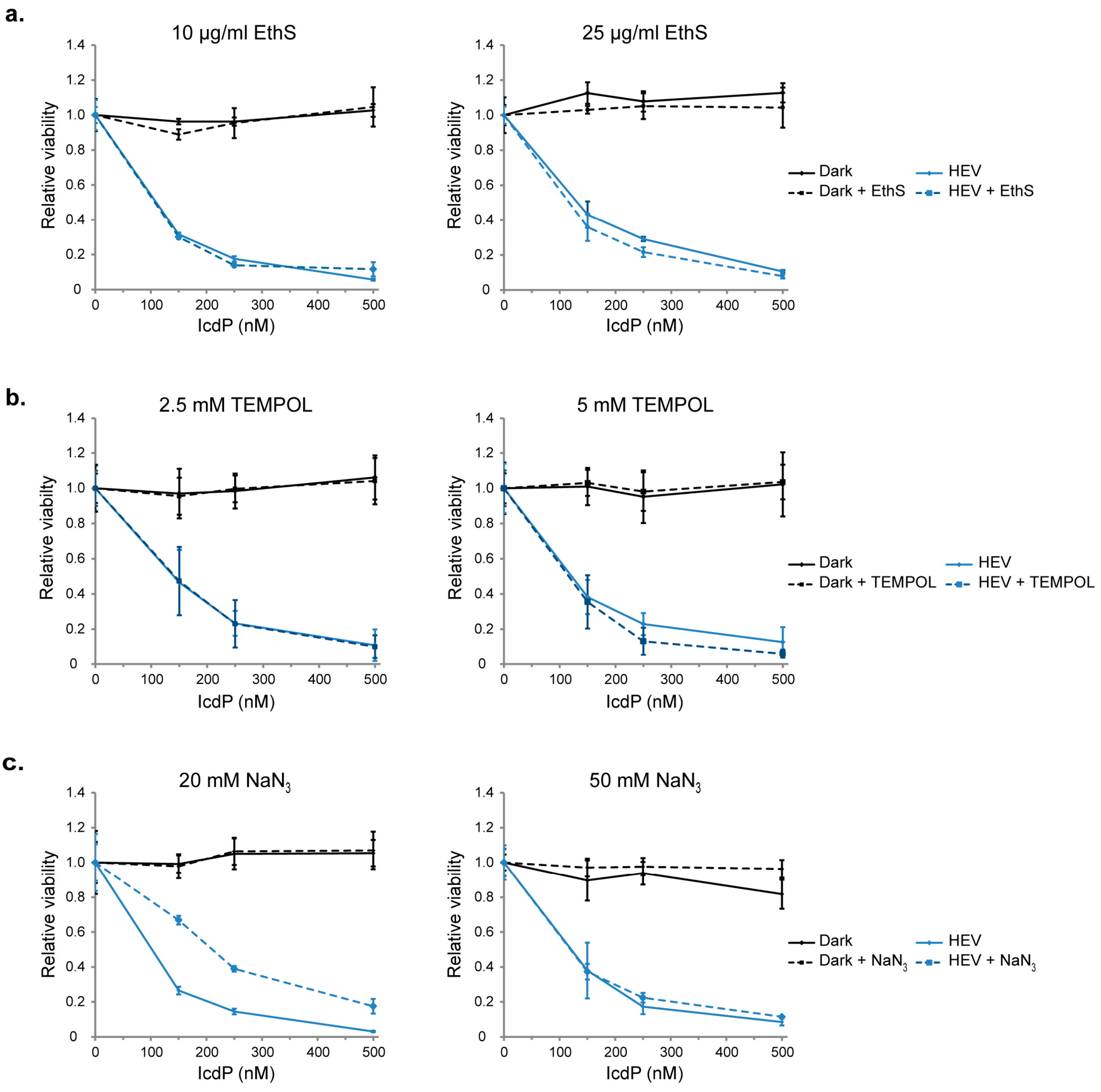

Figure S1: Increasing concentrations of quencher or ROS inhibitors do not provide a better protection against IcdP/HEV-induced loss of viability for RPE cells. ARPE19 cells were treated for 18-24 h with **a)** 10 or 25 µg/ml of ethyl sorbate (EthS), **b)** 2.5 or 5 mM of 4-hydroxy-1-oxyl-2,2,6,6-tetramethylpiperidine (TEMPOL), or **c)** 20 or 50 mM of sodium azide (NaN_3), prior to exposure to IcdP (0 – 500 nM) and/or HEV light (160 J/cm^2). Cell viability was measured 24 h post-exposure using the MTS assay as described in "Materials and Methods". Error bars are SD from quadruplicate assessments (n=4).

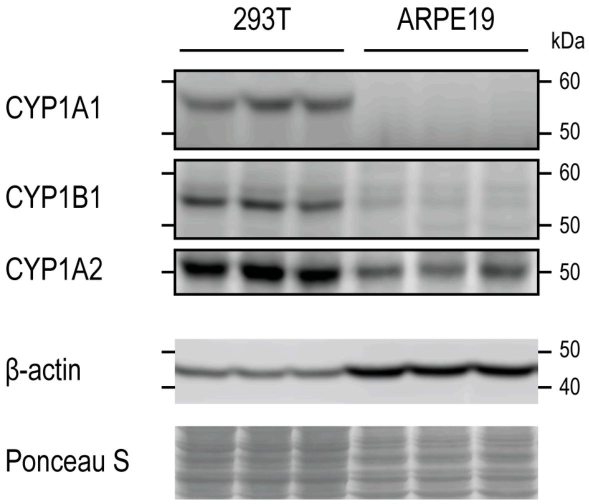

Figure S2: CYP1A1 and CYP1B1 protein levels are undetectable by western blot in ARPE19 cells. Whole protein extract (40 μg) from human embryonic kidney 293T or from ARPE19 cells were subjected to SDS-PAGE western blotting using anti-CYP1A1, anti-CYP1B1 or anti-CYP1A2 antibodies. All CYP1 proteins level can be detected in 293T cells, but only CYP1A2 is observable in ARPE19 cells.

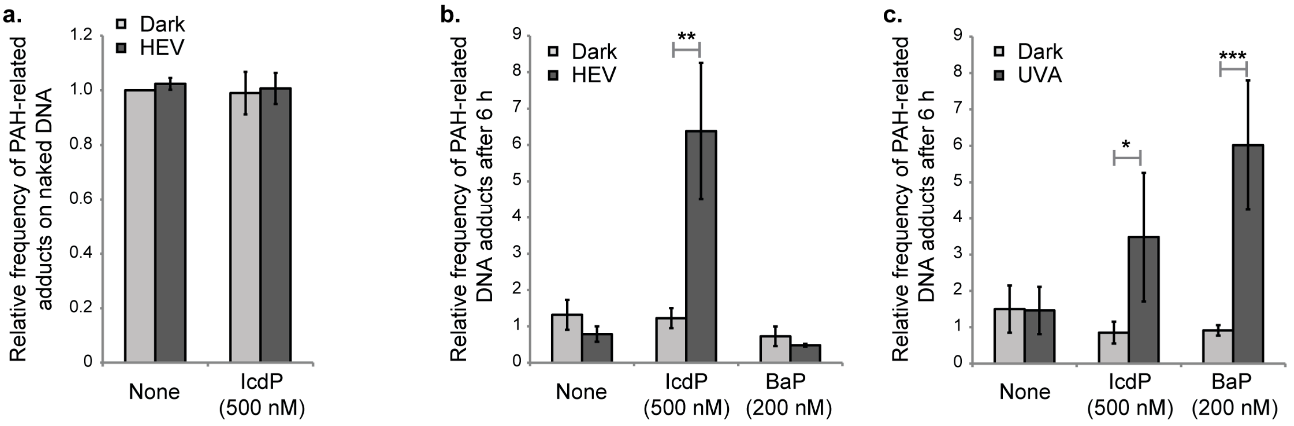

Figure S3: Induction of PAH-DNA bulky adducts by PAH and/or light exposure. a) Naked DNA was exposed *in vitro* to IcdP (500 nM) and/or HEV light (160 J/cm^2). After exposure, the relative frequency of polymerase-blocking bulky adducts directly produced in DNA was assessed using the modified LA-QPCR assay. **b-c)** ARPE19 cells were exposed to IcdP (500 nM) or to BaP (200 nM) in presence or not of **(b)** HEV light (160 J/cm^2) or **(c)** 5 J/cm^2 of UVA radiations along with HEV light (60 J/cm^2). The accumulation of polymerase-blocking bulky adducts in their DNA was assessed 6 h following exposure. For each test, at least 3 independent samples were analysed and each sample processed in quadruplicate. Error bars are SEM; * $p < 0.05$, ** $p < 0.01$, *** $p < 0.001$ [One-way analysis of variance (ANOVA) with Sidak's procedure as post hoc test]
